# Molecular diagnosis for the novel coronavirus SARS-CoV-2: lessons learnt from the Ghana experience

**DOI:** 10.4314/gmj.v54i4s.12

**Published:** 2020-12

**Authors:** Ivy A Asante, Mildred Adusei-Poku, Humphrey K Bonney, Evelyn Y Bonney, John K Odoom, Evangeline Obodai, James Aboagye, Erasmus N Kotey, Stephen Nyarko, Linda Boatemaa, Vanessa Magnusen, Helena Lamptey, George B Kyei, William K Ampofo

**Affiliations:** 1 Department of Virology, University of Ghana, Noguchi Memorial Institute for Medical Research, Legon, Ghana; 2 Department of Medical Microbiology, University of Ghana Medical School, Accra, Ghana; 3 Department of Immunology, University of Ghana, Noguchi Memorial Institute for Medical Research, Legon, Ghana

**Keywords:** SARS-CoV-2, molecular diagnosis, “pooling”, Ghana

## Abstract

**Background:**

A novel coronavirus, SARS-CoV-2 is currently causing a worldwide pandemic. The first cases of SARS-CoV-2 infection were recorded in Ghana on March 12, 2020. Since then, the country has been combatting countrywide community spread. This report describes how the Virology Department, Noguchi Memorial Institute for Medical Research (NMIMR) is supporting the Ghana Health Service (GHS) to diagnose infections with this virus in Ghana.

**Methods:**

The National Influenza Centre (NIC) in the Virology Department of the NMIMR, adopted real-time Polymerase Chain Reaction (rRT-PCR) assays for the diagnosis of the SARS-CoV-2 in January 2020. Samples from suspected cases and contact tracing across Ghana were received and processed for SARS-CoV-2. Samples were ‘pooled’ to enable simultaneous batch testing of samples without reduced sensitivity.

**Outcomes:**

From February 3 to August 21, the NMIMR processed 283 946 (10%) samples. Highest number of cases were reported in June when the GHS embarked on targeted contact tracing which led to an increase in number of samples processed daily, peaking at over 7,000 samples daily. There were several issues to overcome including rapid consumption of reagents and consumables. Testing however continued successfully due to revised procedures, additional equipment and improved pipeline of laboratory supplies. Test results are now provided within 24 to 48 hours of sample submission enabling more effective response and containment.

**Conclusion:**

Following the identification of the first cases of SARS-CoV-2infection by the NMIMR, the Institute has trained other centres and supported the ramping up of molecular testing capacity in Ghana. This provides a blueprint to enable Ghana to mitigate further epidemics and pandemics.

**Funding:**

The laboratory work was supported with materials from the Ghana Health Service Ministry of Health, the US Naval Medical Research Unit #3, the World Health Organization, the Jack Ma Foundation and the University of Ghana Noguchi Memorial Institute for Medical Research. Other research projects hosted by the Noguchi Memorial Institute for Medical Research contributed reagents and laboratory consumables. The funders had no role in the preparation of this manuscript.

## Introduction

### Background

In December 2019, some residents in Wuhan, Hubei province of China, presented to hospitals with pneumonia of unknown origin. Majority of these patients had visited the sea food market in Huanan. Respiratory samples from affected patients were submitted to reference laboratories for investigations. Analyses showed that the pneumonia was caused by a novel coronavirus with >95% homology to bat coronaviruses and >70% similarity with the (Severe Acute Respiratory Syndrome coronavirus) SARS-CoV. The outbreak was consequently reported to the World Health Organization (WHO) on 31^st^ December 2019. The number of cases further increased and spread beyond the Hubei province and subsequently to other countries.^1^,^2^,^3^

The novel virus was initially called the Wuhan coronavirus or 2019 novel coronavirus (2019-nCov) by Chinese researchers.^3^ However, due to its close relatedness to SARS-CoV the International Committee on Taxonomy of Viruses (ICTV) renamed the virus as SARS-CoV-2.^3^,^4^ The WHO named the disease caused by this new virus as Coronavirus disease 2019 (COVID-19). Currently all continents in the world have been affected by this disease with 19 432 244 cases reported worldwide and 721 594 deaths reported to the WHO as at August 9, 2020.^5^ The total number of cases reported by the African continent is 872 501 (4.5% of global cases) with 16 041 reported deaths.^5^,^6^.

Coronaviruses are a large group of viruses that are common among animals. They can be zoonotic and are known to have crossed the species barrier to cause disease in humans as in the Middle East Respiratory Syndrome (MERS CoV).^7^ They can cause mild infections like the common cold to serious infections such as pneumonia and bronchitis.^7^ The incubation period for SARSCoV-2 ranges from 2–14 days.^8^ The virus is spread from human to human through contacts or respiratory droplets through coughs and sneezes. The virus can also be transmitted by touching something an infected person has touched and then touching the mouth, nose or eyes. Signs and symptoms of COVID-19 include fever, cough, sore throat and difficulty breathing. There is currently no vaccine against SARS-CoV-2.^9^ Infection can be prevented by hand washing with soap under running water before touching anything including eyes, nose and mouth, covering mouth and nose when coughing or sneezing, and disinfecting objects and surfaces that are frequently touched. 9

Diagnostic assays include but not limited to nucleic acid amplification tests (NAATs) and rapid antigen (Ag) detection kits.^10^ The NAATs are recommended because they target and amplify viral genomes with higher sensitivity. Rapid diagnostic tests based on Ag detection are currently being developed and commercialized.^10^ These are based on detection of viral proteins, with no amplification involved and therefore are less sensitive.^10^ Other diagnostic tests include serological as well as neutralization tests.^10^ Virus growth in cell cultures are not recommended as routine diagnostic procedures because they require specialized biosafety level III laboratories and trained personnel.^10^ Sequencing of SARS-CoV-2 genome could also be performed when available to investigate transmission dynamics of the outbreak.^10^ The WHO's strategy among others is to “interrupt human-tohuman transmission including reducing secondary infections among close contacts and health care workers, preventing transmission amplification events and preventing further international spread”.

Interrupting human-to-human transmission could be achieved through a combination of public health measures. These include rapid diagnosis, isolation of index cases, contact tracing, healthcare setting infection control, along with population level measures like frequent hand washing, use of hand sanitizers, social distancing and wearing of masks. 11

Ghana, a country in West Africa, had previously recorded cases of Human coronaviruses (HCoV) among the general population.^12^ A study conducted in 2012 detected HCoV in 3.5% out of 200 samples tested at the National Influenza Centre (NIC), with HCoV observed more frequently in outpatient samples compared with in-patients.^12^ Ghana, adopted WHOs strategy of rapidly identifying and isolating infected individuals in an attempt to interrupt further human-to-human transmission. The country has been commended by the WHO and others as a model for the control of COVID-19.^13^ Ghana's success so far has been partly due to a robust testing system initiated by the Noguchi Memorial Institute for Medical Research (NMIMR), University of Ghana. Here we share how the NMIMR rose to the challenge to offer molecular diagnosis in Ghana's COVID-19 response, the challenges faced and lessons that other countries can learn from our experiences. The various efforts and activities that enabled the National Influenza Centre (NIC) to roll out an efficient system to help Ghana use its limited rRTPCR infrastructure across the country to test for SARSCoV-2 are described below.

### Preparedness of the National Influenza Centre (NIC)

Prior to the coronavirus pandemic, Ghana had both an influenza pandemic preparedness and Ebola response plans. In response to this novel coronavirus pandemic, the existing plans were quickly modified to suit the current situation by end of January 2020. The process involved identifying key partners in the response chain. Therefore, Port Health Service - GHS, Disease Surveillance Department of the GHS and regional public health laboratories were identified as key elements of the response which needed resourcing.

### Laboratory response

**Figure 1:**
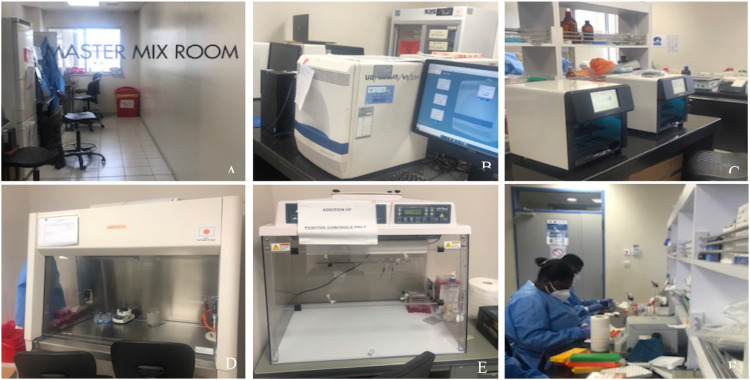
Some selected equipment and processes present at the Advanced Research Laboratory; NMIMR: A: Master Mix room B: Real-Time PCR Machines, C; Genolution automated RNA extraction machine, D: Biosafety Class II A, E: Clean Bench, F: Sample receipt.

The NIC, hosted in the Advanced Research Laboratories at the Virology Department of the NMIMR (Figure 1), undertakes routine surveillance for influenza viruses in Ghana. Since testing for the new COVID-19 virus was based on rRT-PCR protocols, the NIC sourced primers and probes which had been recommended by the WHO (Corman *et al.*, 2020).^14^ Prof. Vincent Munster of the National Institute of Health (NIH), Rocky Mountains, USA on a working visit to the NMIMR facilitated the donation of primers, probes as well as positive controls described by Corman *et al.*, 2020 14 to the NIC.

Trial runs using these primers and probes were successful and the assays were adopted for use. Ghana's NIC was therefore ready to test suspected respiratory specimen for SARS-CoV-2 by the end of January. The Africa Centres for Disease Control and Prevention (Africa CDC) also organized training programs for laboratories in West Africa to build capacity for testing of the new virus. A member of the NIC attended the training and returned with the primers and probes recommended by the WHO for Africa from TIBMOL BIO (Berlin, Germany). The first suspected cases were received by the NIC on February 3, 2020 and the first positive case for Ghana was confirmed on March 12, 2020.

### Case definitions and sample collection

Initially, Ghana strictly followed WHO recommendations for case identification and testing. Suspected cases were defined as:

- A patient with acute respiratory illness (fever and at least one sign/symptom of respiratory disease (e.g., cough, shortness of breath), AND with no other aetiology that fully explains the clinical presentation AND a history of travel to or residence in a country/area or territory reporting local transmission of COVID-19 disease during the 14 days prior to symptom onset; or
- A patient with any acute respiratory illness AND having been in contact with a confirmed or probable COVID-19 case in the last 14 days prior to onset of symptoms or
- A patient with severe acute respiratory infection (fever and at least one sign/symptom of respiratory disease (e.g., cough, shortness breath) AND requiring hospitalization AND with no other aetiology that fully explains the clinical presentation.

Based on the WHO case definitions, suspected cases were initially identified in the hospitals, clinics and health centres. Under this definition, the NIC was receiving about 50 samples per day for testing between February 3 and March 12. Later, the government instituted a policy of ‘enhanced contact tracing’ which did not strictly follow the WHO case definitions. With this, sections of the city were locked down (restriction of human movement) and members of such areas were actively screened for SARS-CoV-2. Under this regime, our centre received approximately 3000 samples per day. The WHO recommends the following samples for COVID-19 diagnosis: Nasopharyngeal and oropharyngeal swab (dacron or polyester flocked), broncho alveolar lavage, (endo) tracheal aspirate, nasopharyngeal or nasal wash/aspirate, sputum, tissue from biopsy or autopsy, including from lung, blood for serum and/or plasma, stool and urine. 10 Irrespective of which sample was collected, all samples were required to be appropriately shipped with triple layer packaging as recommended by the WHO at 4°C (in a cold chain) to the laboratory.

Since expertise for oropharyngeal and nasopharyngeal swab collection already existed in the country, these were the samples of choice for Ghana. The country started with collection of oropharyngeal and nasopharyngeal swabs, but these quickly run out due to the huge numbers of samples. However, because it had been previously shown that sputum, (a lower respiratory tract specimen), yielded equally good amounts of virus, the country switched to sputum collection. 15Sterile sputum containers were readily available. Sputum samples were received in sterile sputum containers and processed. One milliliter of sterile physiological saline (0.9% NaCl solution) was added to sputum and then transferred into previously labelled cryovials.

### Laboratory procedures

The evolving and high turnover nature of the testing required that several assays and procedures be used to cope with the mounting need for testing. The details of these activities are described in detail to highlight the challenges that were encountered.

#### Pooling

**Figure 2:**
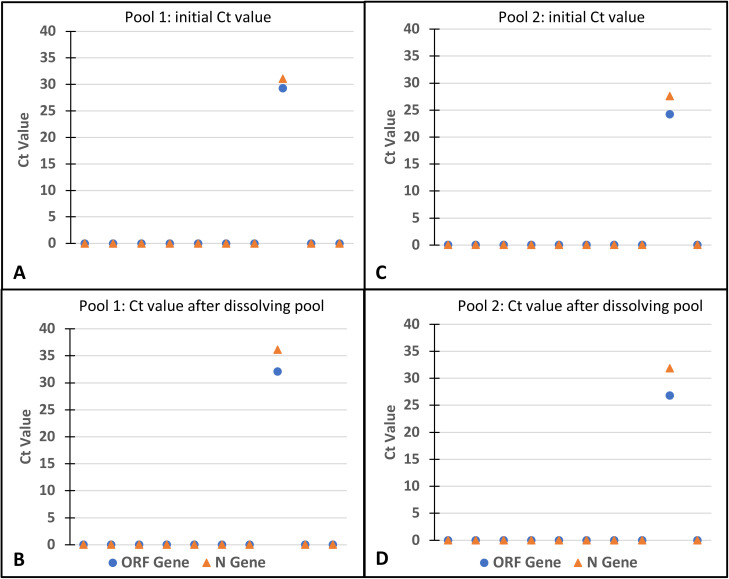
Validation of pooling method prior to adaptation for testing for SARS-CoV-2 at the NMIMR. We pooled twenty samples into two pools of ten individual samples each. Each pool contained a previously known positive sample of varying Ct values. RNA extraction was done using the QIAamp Viral RNA Mini kit (QIAGEN, Hilden, Germany) and SARS-CoV-2 RNA was detected in rRT-PCR assays using the Detection Kit for 2019 Novel Coronavirus (2019-nCoV) RNA (PCR-Fluorescence Probing)’ by Da An Gene Co., Ltd. of Sun Yat-sen University, Guangdong, China. **A**: Initial cycle threshold (Ct) values of positive sample in pool 1 (sample with high Ct value) **B**: Ct value after pool 1 is dissolved **C:** Initial Ct value of positive sample in pool 2 (sample with low Ct value) **D** Ct value after pool 2 is dissolved. Blue circle represents open reading frame (ORF) and orange triangle represents nucleocapsid gene (N gene).

Due to the large numbers of samples (approximately 3000 samples) that were received on a daily basis, samples were ‘pooled’ to facilitate testing. Pooling has been previously described by several authors to facilitate processing of large numbers of samples. ‘Pooling’ involves pipetting equal amount of multiple samples into one tube. The volume of specimen to be pipetted depends on the starting volume for the RNA isolation process being used. This process has become one of the most significant ways for increasing testing of large numbers of samples, especially due to shortages in reagents needed for the rRT-PCR. Pooling is highly efficient and saves time and reagents. 16–19 Pooling was first validated before it was applied for diagnostics. To validate the method, equal volumes of known negative samples were taken into a new tube and the same volume of one known positive sample was added, mixed and RNA isolated from the mixture using the QIAamp viral RNA extraction kit (QIAGEN, Hilden, Germany) following the manufacturer's instructions. Two categories of positive samples were used: samples with low positivity (Ct value of ≥35) and highly positive samples (Ct value ≤25) (Figure 2). Isolated RNA from the pools were subject to rRT-PCR using Detection Kit for 2019 Novel Coronavirus (2019-nCoV) RNA (PCR-Fluorescence Probing)’ by Da An Gene Co., Ltd. of Sun Yat-sen University, Guangdong, China. SARS-CoV-2 RNA was detected in both pools an indication that positive samples will still be detected in a mix of negative samples when they are pooled together. Subsequently, samples were pooled in 10s (and later 5s) and pools tested for the presence of SARS-CoV-2 RNA. Any pool that tested positive was dissolved and the individual samples tested to determine which sample/samples tested positive. The various volumes of samples ‘pooled’ depending on the RNA extraction kit available is shown in Table 1.

**Table 1:** Volumes of samples pooled depending on RNA extraction kit available

RNA Extraction Kit	Volume of sample in a pool (µL)	Total volume of sample/ pool (µL)
**QIAamp Viral RNA Minikit (QIAGEN, Hilden,** **Germany)**	14	140
**Beaver Nucleic Acid Extraction kit (Beaver Biomedical** **Engineering Co. Ltd., Jiangsu, China)**	20	200
**Zymo Quick-RNA Miniprep kit (Zymo Research** **Corporation, California, United States of America)**	25	250
**RNeasy kit (QIAGEN, Hilden, Germany)**	14	140
**DAAN Gene RNA Extraction kit (DAAN gene** **Co. Ltd., Guangdong, China)**	20	200
**NX-48S Viral RNA Extraction kit (Genolution)** **(Genolution Pharmaceuticals Inc., Seoul, Soth** **Korea)**	20	200
**Viral Nucleic Acid Extraction kit II (Geneaid)** **(Geneaid Biotech Ltd., New Taipei, Taiwan).**	20	200

#### RNA isolation

RNA isolation was done based on the availability of a particular kit following the manufacturer's protocols. As the laboratory rapidly run out of reagents and consumables, several kits were used for RNA isolation as they became available. The initial kit used was the QIAamp viral RNA Minikit (QIAGEN, Hilden, Germany). This kit, based on silica membrane technology is used for rapid isolation of high-quality, ready-to-use RNA.^20^ When the laboratory run out of this kit, nucleic acid extraction kit from Beaver 21 (Beaver Biomedical Engineering Co. Ltd., Jiangsu, China) was used. Beaver nucleic acid extraction kit is used to rapidly and efficiently extract viral genomic RNA/DNA from cell-free body fluids. Other RNA isolation kits that were used included the Zymo Quick-RNA Miniprep kit (Zymo Research Corporation, California, United States of America), RNeasy (QIAGEN, Hilden, Germany), RNA extraction kits from DAAN gene Co. Ltd., Guangdong, China donated by Jack Ma, the Chinese billionaire, NX-48S Viral RNA kit from Genolution Pharmaceuticals Inc., Seoul, South Korea and Viral Nucleic Acid Extraction Kit II from Geneaid Biotech Ltd., New Taipei, Taiwan.

Table 2 outlines the starting materials needed for these RNA isolation kits as well as the principles behind their use. The performance of each kit was assessed based on equipment requirement, labour intensiveness and turnaround time. Finally, our laboratory settled on three commercially available extraction kits. These were the Viral Nucleic Acid Extraction kit II from Geneaid, the RNA extraction kit from DAAN Gene Co. Ltd., which were donated by Jack Ma, and the NX-48S Viral RNA kit from Genolution in combination with the automated Genolution extraction machine. Currently our laboratory has three of the Genolution automated machines.

**Table 2:** RNA isolation kits and the mode of operations

RNA extraction kit	Starting Material	Principle
**QIAamp viral RNA Minikit (QIAGEN, Hilden, Germany)**	Cell-free body fluids	Viral RNA binds specifically to the QIAamp silica membrane and is eluted with water or an elution buffer provided
**Beaver nucleic acid extraction kit (Beaver Biomedical** **Engineering Co. Ltd., Jiangsu, China)**	Cell-free body fluids	Use of super paramagnetic microspheres and without centrifugation, using prefabricated buffers allows direct extraction
**Zymo Quick-RNA Miniprep kit** **(Zymo Research Corporation, California, United States** **of America)**	Wide range of cells (up to 10^7^) and tissue samples	Procedure combines a unique buffer system with Zymo-Spin• column technology
**RNeasy kit (QIAGEN, Hilden, Germany)**	Cells, tissues, and yeast	Silica-membrane RNeasy spin columns with a binding capacity of 100 µg RNA
**RNA Extraction kit (DAAN gene Co. Ltd., Guangdong,** **China)**	Cell-free body fluids	Column technology with the use of proteinase K
**NX-48S Viral RNA Extraction kit (Genolution Pharmaceuticals** **Inc., Seoul, Soth Korea)**	Cell-free body fluids	An automated protocol that works on the Genolution automated RNA extraction machine.
**Viral Nucleic Acid Extraction kit II (Geneaid Biotech** **Ltd., New Taipei, Taiwan).**	Wide variety of viruses.	Column purification utilizing an efficient glass fiber spin column system

#### Real-Time Reverse Transcription-Polymerase Chain Reaction (rRT-PCR)

The use of rRT-PCR kits and primers followed the same pattern as viral RNA isolation kits. Kits were used as and when they were available. Initially, primers and probes described by Corman *et al.*, 2020,^14^ which had been adopted by the WHO were used. The assay was designed to target the E gene of SARS CoV as well as the discriminatory RNA dependent RNA polymerase of the 2019 SARS-CoV-2 virus. The 25-µl reaction assay was set up to contain 12.5µl of 2X reaction buffer provided with the Superscript III one step RT-PCR system with Platinum Taq Polymerase (Invitrogen; containing 0.4 mM of each deoxyribonucleotide triphosphates (dNTP) and 3.2mM magnesium sulfate), 1µl of reverse transcriptase/Taq mixture from the kit, 0.4µl of a 50mM magnesium sulfate solution (Invitrogen – not provided with the kit), 1µg of nonacetylated bovine serum albumin (Roche) and 5µl of RNA. This reaction mix was later halved to save reagents. Cycling conditions were as outlined in Table 3.

**Table 3:** Cycling conditions for various rRT-PCR kits used

PCR Kit: TIBMOL BIO (Berlin, Germany)		
Step	Cycling conditions	Number of cycles
**Reverse transcription**	55°C: 10mins	
**Denaturation**	95°C: 3mins	
**Annealing and Extension**	95°C: 15sec, 58°C: 30sec	45
**PCR Kit: 2019-nCoV RNA PCR-Fluorescence Probing kit (Da An gene Co. Ltd., Guangdong, China)**		
**Step**	**Cycling conditions**	**Number of cycles**
**Reverse transcription**	50°C: 15min	
**Denaturation**	95°C: 15mins	
**Annealing and Extension**	94°C: 15sec, 55°C: 45sec	45
**PCR Kit: MiRXES Fortitude Kit 2.1 RUO (MiRXES Pte Ltd., Central Region, Singapore)**		
**Step**	**Cycling conditions**	**Number of cycles**
**Reverse transcription**	48°C: 15min	
**Denaturation**	95°C: 2mins 30sec	
**Annealing and Extension**	95°C: 10sec, 59°C: 42sec	42

Subsequently, Detection Kit for 2019 Novel Coronavirus (2019-nCoV) RNA (PCR-Fluorescence Probing)’ by Da An Gene Co., Ltd. of Sun Yat-sen University, Guangdong, China was received as a donation. This kit is based on one-step RT-PCR technique. Specific primers and fluorescent probes had been designed for the detection of 2019 Novel Coronavirus RNA in given specimens. It also contained an endogenous internal standard detection system which was used for monitoring the processes of specimen collection, RNA and PCR amplification thereby reducing false negative results. This assay required a 25µl reaction volume containing 17 µl of NC (ORF 1ab/N) PCR reaction solution A (main constituents: specific primers, probes, tris (hydroxymethyl) aminoethane-hydrochloric acid buffer), 3µl of NC (ORF 1ab/N) PCR reaction solution B (main constituents: Hot start Taq DNA polymerase, C-MMLV reverse transcriptase) and 5µl template i.e. patient RNA as well as positive and/ or negative controls. Cycling conditions were as provided in Table 3.

The reaction volumes were halved in order to save reagents. Again, the lab used the MiRXES Fortitude Kit 2.1 RUO (MiRXES Pte Ltd., Central Region, Singapore). This was a real-time reverse transcriptase polymerase chain reaction (RT-PCR) assay, for the quantitative detection of SARS-CoV-2 specific RNA in nasopharyngeal swab samples. The assay requires a 25µl reaction volume which should contain 5.9µl nuclease-free water, 12.5µl Universal Probes Reaction Mix, 1.0µl Primers-Probe Mix 2.1, 0.5µl Reverse Transcriptase, 0.1µl Internal Control as well as 5µl of either Extracted RNA, positive or negative control. The cycling conditions are provided in Table 3. Again, reaction volumes were halved to save reagents. Therefore, the RT-PCR kit used depended on what was available in the laboratory and all reagents were halved.

### Collaboration

**Figure 3:**
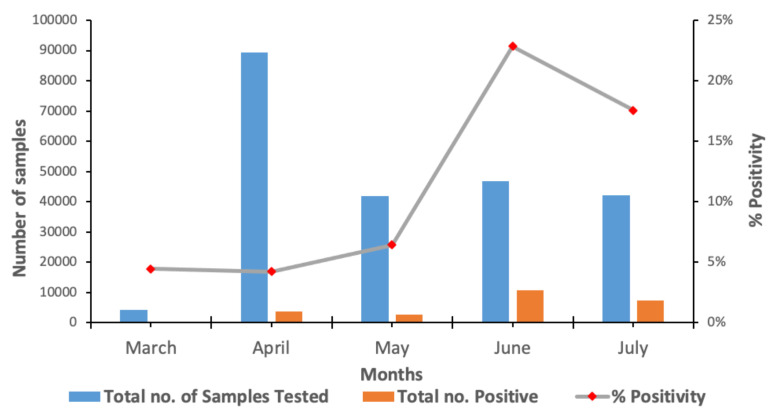
Distribution of SARS-CoV-2 cases across months tested in Ghana

Several factors have contributed to the successes achieved by Ghana so far. These include previous diagnostic capacity for testing for influenza and other respiratory viruses at the NIC, availability of experienced virologists with extensive knowledge in molecular work, research assistants from virology staff with good working knowledge in molecular techniques, newly trained National Service Personnel, technicians with extensive experience in working with various equipment and data entry clerks. In addition to the human resources were the state-of-the-art infrastructure which included pathogen level III containment laboratories and equipment (Figure 1). Expertise was readily available at the NMIMR. Members of the institute were called upon to assist the NIC with testing large number of samples. Again, volunteers from the GHS, staff from private labs, staff from other schools and centres of the University of Ghana including the West African Centre for Cell Biology and Infectious Pathogens (WACCBIP), the University of Ghana Medical School, School of Allied Health Sciences as well as staff from the Ghana Association of Medical Laboratory Scientists (GAMLS). Three teams of approximately 40 members each were then formed, each led by experienced and dedicated Virologists. These teams worked twelve-hour shifts, ensuring that testing was available 24 hours. Due to dedicated and self-less service, the laboratory at the NMIMR has tested a total of 283 946 samples as at August 21, 2020 (Table 4). Out of this number, 10% (28 736/283 946) have been positive for SARS-CoV-2, with the highest positivity rate seen in the month of June (Figure 3).

**Table 4:** Number of samples tested at the NMIMR for COVID-19 from February 3 to August 4, 2020

Category of samples tested	Total number of samples	Number Positive (%)
**Hospitals (clinical samples)**	144 783	20 796 (14.3%)
**Hotels (initial)**	1 045	79 (7.6%)
**Hotels (exit)**	966	26 (2.7%)
**Contact tracing**	137 152	7 835 (5.7%)
**Total**	**283 946**	**28 736 (10.1%)**

Samples received from various hospitals are categorized as Hospitals (clinical samples). Travelers who were initially quarantined when government announced closure of the airport are labelled Hotels (initial) and their exit results as Hotels (exit). Samples from aggressive contact tracing efforts are labelled Contact Tracing.

### Challenges

The capacity of the NIC to handle large amounts of samples did not come without challenges. These need to be examined to inform the development for diagnosis for the entire country.

#### Rapid use of reagents and consumables

The COVID-19 laboratory at the institute quickly run out of reagents and consumables to use due to their rapid use. However, being part of a large research institute, it was possible to borrow laboratory consumables and reagents from other projects that were at a standstill due to the pandemic. The major global effect of pandemics on logistics will require strategic stockpiling as well as possible local production and rapid high level decision making to ensure access and provision of essential laboratory supplies to support public health responses.

#### Lack of uniformity with nucleic acid isolation

The COVID laboratory used whatever RNA isolation kit was available. This may have led to non-uniformity in testing. However, each new RNA isolation kit was assessed before it was used, and this may have mitigated variations. In the future, the laboratory may settle on a list of kits that it already assessed and evaluated.

#### Data management

A proper laboratory data management system was initially not available for receipt of thousands of samples daily. A simple Microsoft Excel software program was used to manage data per the GHS case-based form that captured information on suspected COVID-19 cases. The GHS eventually established the online **S**urveillance Outbreak Response Management Analysis System (SORMAS) with electronic barcoding which has improved data management.

#### Lack of timely adequate funding

Testing for SARS-CoV-2 is done by the NMIMR as specialized diagnostic test to support the GHS. However, lack of timely adequate funding for procurement of laboratory supplies, hiring of staff and maintenance of equipment were severe bottlenecks. We therefore resorted to borrowing from other research projects in Virology and other Departments of the Institute. Thus, we almost depleted the Noguchi stores of all items needed for COVID testing before the National supplies started coming in.

#### Sample packaging

There were issues with respiratory samples submitted for testing. These included sample leakages, mismatched samples, improper labelling and unsuitable sample collection swabs and sputum containers. This led to some samples being rejected and discarded. Subsequently Standard Operating Procedures were developed and adopted for sample receipt.

#### Operational Issues

In addition to the challenges outlined above, there were some operational challenges such as ensuring rapid daily processing of thousands of samples during the lock down period which led to back-logs of samples. Lack of adequate storage for >200,000 samples led to problems with real time identification and retrieval which are critical steps in the ‘pooling’ strategy.

### Lessons learnt

Several lessons have been learnt while responding to this pandemic. The most important one being adequate preparation, planning, resource allocation and manpower training. Every country including Ghana needs an infectious disease pandemic preparedness plan. This plan should clearly outline what needs to be done at every stage. One of the strong points for Ghana was the availability of a Pandemic Influenza Preparedness plan and an active NIC at the NMIMR well versed in laboratory diagnosis of respiratory viruses. Another strong point that worked for Ghana is collaboration. Over the years, the NMIMR had established several collaborations with several partners including the GHS, WHO US NAMRU#3 Ghana detachment, US CDC, Africa CDC, Japan International Corporation Agency (JICA) amongst others. Together with local organizations including Banks and Mining companies, they gave support in the form of laboratory reagents and consumables. Again, various Departments in the Institute provided expertise and manpower to support testing. The most important lessons are adequate planning, resource mobilization, data management and collaboration. These are salient for an effective response to a pandemic.

## Conclusion

The NMIMR is currently the largest SARS-CoV-2 testing centre in Ghana. This has been due to the presence of well-established strong collaboration with the GHS/Ministry of Health, the WHO, and the strong internal networks in the Institute. The institute has been well supported by its partners as well as the Government of Ghana. The availability of the new Advanced Research Laboratory center recently provided by the Government of Japan provided critical infrastructure and equipment to handle the large volume of testing. The Institute is currently strengthening all elements of its quality management system to enhance the services it provides to the nation. Testing for SARS-CoV-2 in Ghana at the NMIMR, University of Ghana, has been largely successful despite the challenges.
